# Comparison of single and double chest drains following pulmonary lobectomy

**DOI:** 10.1371/journal.pone.0319077

**Published:** 2025-05-08

**Authors:** Ahmed Elmezayen, Ahmed Osama, Amal Said Elbendary, Abdelrahman Abdelbar

**Affiliations:** 1 Cardiothoracic Surgery Department, Faculty of Medicine, Tanta University, Tanta, Egypt. Basildon University Hospital, London, United Kingdom; 2 Pediatrics Department, Mabara Hospital, Ministry of Health, Tanta, Egypt; 3 Clinical Pathology Department, Faculty of Medicine, Tanta University, Tanta, Egypt; 4 Cardiothoracic Surgery Department, Manchester University NHS Foundation Trust, Manchester, United Kingdom; European Institute of Oncology: Istituto Europeo di Oncologia, ITALY

## Abstract

**Background:**

Chest tubes are commonly used to empty the chest cavity after pulmonary lobectomy. Although two drains have traditionally been used to guarantee proper air and fluid evacuation, they frequently cause patients more pain and lengthen their hospital stays. This study set out to compare the effectiveness of using a single chest drain versus using two chest drains after a pulmonary lobectomy.

**Methods:**

This retrospective trial was performed on 50 patients aged ≥18 years, both sexes, scheduled for Video-Assisted Thoracic Surgery (VATS) lobectomy. Patients were divided into two equal groups: Group S: single chest tube was used and Group D: double chest tubes were used.

**Results:**

The duration of drainage was 3.32 ± 0.69 days in group S and was 4.2 ± 1.29 days in group D (P < 0.05). The amount of drainage was 593.64 ± 45.94 ml in group S and was 910.04 ± 71.42 ml in group D (P < 0.05). Assessment of the pain using the visual analog scale on second day was insignificantly different between both groups and was significantly lower at the second postoperative week in group S than in group D (P = 0.005). Length of hospital stays and complications (pneumonia, re-drainage, and persistent air leak (> 7 days)) were insignificantly different between both groups.

**Conclusions:**

The effectiveness of inserting one chest tube following a pulmonary lobectomy is comparable to that of inserting two tubes. Furthermore, employing a single tube is more advantageous than two tubes, as it is associated with lower postoperative pain, duration and amount of drainage.

## Introduction

Pulmonary lobectomy is a thoracic surgery that is essential for the management of a variety of lung diseases, such as lung cancer, benign lung tumors, and infectious diseases [1].

Pulmonary lobectomy can lead to postoperative complications including fluid collection, pneumothorax, air leaks, and decreased lung function. [2,3]. A chest drain is typically inserted at the conclusion of the surgical process to deal with these possible complications [4]. There is still some debate among thoracic surgeons as to whether a single or double drain is better for the chest following a pulmonary lobectomy [5].

The pleural area is typically drained using a pair of chest tubes after a resection of the lung [6]. One is inserted into the basilar area and the back, and to remove any trapped air, the other is guided towards the apex to collect fluid [7]. Primarily, it was believed that two chest tubes were necessary to keep the pleural region completely sealed off from air and fluid [8]. Two chest tubes post-pulmonary resection raise the chance of atelectasis and collapsed lung, two postoperative problems that are already present. There is a higher chance of pain during the procedure, which can have a major impact on postoperative respiration and chest wall compliance [9].

Some studies have demonstrated that after resections, a single chest tube may be sufficient [7,10]. In theory, a single chest tube should be less invasive and more cost-effective than two during the postoperative period. Postoperative pain reduction has the potential to enhance the patient’s capacity to engage in respiratory activities, leading to enhanced lung expansion and reduced complication rates. An earlier discharge from the hospital is another possible outcome [11].

The present study set out to evaluate the relative merits of using a single chest drain following pulmonary lobectomy compared to two separate drains.

### Patients and methods

This retrospective study involved 50 patients aged ≥18 years, both sexes, scheduled for lobectomy. The research was conducted from September 2022 to February 2023 following approval from the Ethical Committee Tanta University Hospitals, Egypt (approval code: 36264PR101/08/22). The patients were divided into two groups: Group S: a single chest tube was used and Group D: double chest tubes were used.

Exclusion criteria were pregnant patients, symptoms of shock (low blood pressure, changes in mental status, etc.), and perioperative massive air leakage or massive bleeding.

The patient underwent a thorough evaluation that included taking a medical history, having a physical examination, conducting laboratory investigations, and measuring electrolytes. Electrocardiograms with 12 leads were used as necessary. Whenever necessary, chest X-rays and CT scans were acquired. Finally, preliminary assessments were conducted by the anesthesiology team. In this series we included only patients who had lung resections for lung cancer, and we didn’t include cases with infective etiology. ERAS protocol was used.

### Surgical method

Video-Assisted Thoracic Surgery (VATS) procedures were typically conducted under general anesthesia in a lateral decubitus position by three consultant thoracic surgeons, utilizing either a double or single endotracheal tube for single-lung ventilation.

Following hemostasis, the control group underwent the insertion of two 32 F polyethylene chest tubes. One was situated along the middle axillary line on the side of the chest that was more reliant, and the other was located along the front axillary line near the upper part of the chest.

In contrast, patients subjected to the single tube technique had one chest tube of the same diameter inserted in the midaxillary line of the hemithorax’s dependent side, which was then routed towards the apex. Neither group actively reduced the chest cavity using techniques like pleural tenting or pneumoperitoneum creation. Upon completion of the procedure, chest tubes were placed.

### Postoperative care

As part of their treatment, every patient was required to spend at least an hour in the recovery room. The chest tubes were attached to a seal that was submerged. Each chest tube was connected to one of two individual drainage bottles in the control group. Measures were putting the drains on suction intermittently with negative pressure (15–20 cm H_2_O) till air leak stopped and proper chest physiotherapy using an incentive spirometer from day one postoperatively and this was adequate to drain fluid and air

An incentive spirometer was used to motivate patients to cough. If there were no contraindications, patients could be readmitted to the thoracic surgical unit the very same day. Documentation of air leakage and the quantity of drainage was done twice daily. A chest X-ray was conducted on a daily basis until the day of discharge and again prior to the removal of the chest tube(s).

The initial air leak stopped after 2 days but we faced 10 cases with prolonged air leaks (4 in group S and 6 in group D) that need reinsertion of the drain and aggressive physiotherapy. Once the air leak stopped, the chest tubes were expelled from the single tube group and pleural drainage dropped to less than 200 cc/day. The posterior tube was withdrawn from the control group as soon as the daily drainage from the pleura dropped below 200 cc. A residual pleural gap can still be eliminated if there is no air leak. After every patient had their chest tubes removed, a chest X-ray was taken. Assuming no complications occurred, all patients were given permission to depart the hospital one day after the chest tubes were removed.

### Pain management

On the first postoperative day, patients were administered a patient-controlled analgesic consisting of morphine (2 mg loading dose, 1 mg bolus with a 30-minute lock-out time). The standard analgesic regimen for all patients was 1 gram of paracetamol every 6 hours.

Before the analgesic was administered on the second morning after surgery, the level of pain was measured using a visual analog scale (VAS). zero labeled “no pain” and ten labeled “worst pain imaginable.” The line is 10 cm in length. On this line, the patients were instructed to indicate the level of pain they were experiencing. Before surgery, patients learned about VAS and its grading system. They came to our clinic two week after leaving the hospital with a prescription for nonsteroidal anti-inflammatory drugs (NSAIDs).

The primary outcome was the duration of chest tube drainage. The secondary outcomes were the amount of drainage, pain score, duration of hospital stay, and complications.

### Sample size calculation

G*Power 3.1.9.2 (Universitat Kiel, Germany) was employed to conduct the sample size. According to a previous study [12], the mean ± SD of the duration of drainage was 3.4 ± 1.01 days with single-tube group and 4.4 ± 0.9 days with double-tube group. We enrolled 25 patients in each group based on a 95% confidence limit, 90% power, an allocation ratio of 1:1, and an effect size of 1.045 in the study and to accommodate for possible dropouts, each group received four more cases.

## Statistical analysis

SPSS v27 (IBM©, Armonk, NY, USA) was applied to conduct the statistical analysis. The data was examined for normality of distribution using histograms and the Shapiro-Wilks test. The unpaired student t-test was employed for analyzing quantitative parametric data, which was reported as mean and standard deviation (SD). The Mann Whitney U test was employed to examine quantitative non-parametric data in order to find the median and interquartile range (IQR). We utilized either Fisher’s exact test or the Chi-square test to examine the qualitative variables, which were presented as percentages and frequencies, respectively. To prove statistical significance, the two-tailed P value has to be less than 0.05.

## Results

Both groups did not differ significantly with respect to demographic data, parenchyma staple length, or pleural sealant use. Table 1

**Table 1:** Demographic data, length of the parenchyma staples, and use of pleural sealants of the studied groups.

		Group S (n = 25)	Group D (n = 25)	P value
**Age (years)**		47.04 ± 10.82	49.24 ± 12.83	0.515
**Sex**	**Male**	19 (76%)	18 (72%)	0.747
	**Female**	6 (24%)	7 (28%)	
**Laterality**	**Right**	14 (56%)	17 (68%)	0.382
	**Left**	11 (44%)	8 (32%)	
**Resected lobe**	**Upper**	8 (32%)	7 (28%)	0.838
	**Middel**	4 (16%)	3 (12%)	
	**Lower**	13 (52%)	15 (60%)	
**Pleural adhesions**		9 (36%)	11 (44%)	0.564
**Length of the parenchyma staples (cm)**		7.6 ± 1.47	8.32 ± 1.7	0.116
**Use of pleural sealants**		4 (16%)	3 (12%)	1

Data are presented as mean ± SD or frequency (%).

The duration of drainage was 3.32 ± 0.69 days in group S and was 4.2 ± 1.29 days in group D. The amount of drainage was 593.64 ± 45.94 ml in group S and was 910.04 ± 71.42 ml in group D. Group S had significantly lower duration and amount of drainage than in group D (P = 0.004, P < 0.001 respectively). **Table 2.**

**Table 2:** Duration of drainage and amount of drainage of the studied groups.

	Group S (n = 25)	Group D (n = 25)	P value
**Duration of drainage (days)**	3.32 ± 0.69	4.2 ± 1.29	0.004*
**Amount of drainage (ml)**	593.64 ± 45.94	910.04 ± 71.42	<0.001*

Data are presented as mean ± SD. *: Significant when P value ≤ 0.05.

Neither group differed significantly from the other on VAS on the second day. Group S had a significantly lower VAS at the second week than group D. (P = 0.005). **Table 3**.

**Table 3:** VAS of the studied groups.

	Group S (n = 25)	Group D (n = 25)	P value
**Second day**	2 (2-3)	3 (2-3)	0.157
**Second postoperative week**	1 (0-1)	1 (1-2)	0.005*

Data are presented as median (IQR). *: Significant when P value ≤ 0.05. VAS: Visual analog scale.

Complications (pneumonia, pleural empyema, wound infection, pneumothorax required re-drainage, hemothorax required re-drainage, prolonged air leak and second drain insertion) and hospital stays were insignificantly different between both groups. **Table 4**

**Table 4:** Complications and hospital stays of the studied groups.

		Group S (n = 25)	Group D (n = 25)	P value
**Complications**	**Pneumonia**	1 (4%)	2 (8%)	1
	**Pleural empyema**	0 (0%)	1 (4%)	1
	**Wound infection**	1 (4%)	0 (0%)	1
	**Pneumothorax required** **re-drainage**	1 (4%)	2 (8%)	1
	**Hemothorax required** **re-drainage**	1 (4%)	1 (4%)	1
	**Persistent air leak** **(> 7 days)**	4 (16%)	6 (24%)	0.725
**Hospital stays (days)**		4.04 ± 1.06	4.32 ± 1.22	0.390

Data are presented as frequency (%)

## Discussion

The failure of the residual lung to adequately expand is a prevalent consequence following large anatomical resections such as lobectomy [13]. The majority of thoracic surgeons and textbooks advise placing two drains following these surgeries to circumvent this issue. As a matter of course, one drain should be placed in front and apically, and the other back and basally. Following this advice ensures that the pleural cavity is adequately evacuated of air and fluids (blood or exudations). After lobectomy, we thought that one correctly placed drain would be enough because the mediastinum and diaphragm are displaced, therefore the margins of the pleural space are not established [10].

According to our findings, the group with only one chest tube had far less drainage and a shorter duration than the group with two tubes. VAS on the second day was insignificantly different between both groups. VAS at the second postoperative week was significantly lower in the single chest tube group than in the double chest tube group. Complications and hospital stays were insignificantly different between both groups.

The results of Alex et al. [14] corroborate our own, showing that the two groups did not significantly differ in terms of time of drainage, amount of stay, or amount of drainage. In terms of pain score, the group with two chest tubes was significantly more affected than the one with one.

Okur et al. [15] observed that total pleural drainage was significantly lower in the group that had only one tube compared to the group that had two tubes. There was a slight reduction in early and late postoperative pain for patients whose surgeries only required a single tube. The single-tube group required less time to empty their chest tubes and spent less time in the hospital. Neither group had noticeably higher or lower rates of problems than the other.

In addition, Pawelczyk et al. [12] indicated that a only one chest tube is enough to shorten the duration of hospitalization following lobectomy.

In contrast to our findings, Gomez-Caro et al. [16] conducted research and discovered that both groups had comparable amounts and duration of drainage. Tanaka et al. [17] revealed no significant difference between both groups’ drainage amounts and durations and pain levels.

This study has a few limitations, such as retrospective nature, small sample size, using a single-center design, and a short follow-up period for patients. Therefore, we suggested conducting more studies with more extensive follow-up periods and a larger sample size.

## Conclusions

The effectiveness of inserting one chest tube following a pulmonary lobectomy is comparable to that of inserting two tubes. Furthermore, employing a single tube is more advantageous than two tubes, as it is associated with lower postoperative pain, duration and amount of drainage.

## Supporting information

S1 Data Master sheet. (XLSX)

## References

[1] Harmouchi H, Lakranbi M, Issoufou I, Belliraj L, Ammor F, Rabiou S. Surgical outcomes of pulmonary lobectomies for malignant diseases: Experience of a moroccan center about 42 cases. Clin Surg. 2019;2360:612–87.

[2] Geraci TC, Chang SH, Shah SK, Kent A, Cerfolio RJ. Postoperative air leaks after lung surgery: predictors, intraoperative techniques, and postoperative management. Thorac Surg Clin. 2021;31(2):161–9. doi: 10.1016/j.thorsurg.2021.02.005 33926669

[3] Leivaditis V, Skevis K, Mulita F, Tsalikidis C, Mitsala A, Dahm M, et al. Advancements in the management of postoperative air leak following thoracic surgery: from traditional practices to innovative therapies. Medicina (Kaunas). 2024;60(5):802. doi: 10.3390/medicina60050802 38792985 PMC11123218

[4] Lodhia J, Suleman M, Chugulu S, Chilonga K, Msuya D. Chest tube thoracostomy: A simple life-saving procedure with potential hazardous risks. Int J Surg Case Rep. 2023;108:108416. doi: 10.1016/j.ijscr.2023.108416 37343502 PMC10382721

[5] Hart JM, Hussien AM, Tesfaye S, Nadamo SM, Senbu MF, Wadaja DF, et al. Effectiveness of single chest tube vs double chest tube application postdecortication: prospective randomized controlled study. J Am Coll Surg. 2023;236(6):1217–31. doi: 10.1097/XCS.0000000000000661 36808127 PMC10174098

[6] Hassan M, Touman AA, Grabczak EM, Skaarup SH, Faber K, Blyth KG, et al. Imaging of pleural disease. Breathe (Sheff). 2024;20(1):230172. doi: 10.1183/20734735.0172-2023 38482187 PMC10928554

[7] Shintani Y, Funaki S, Ose N, Kanou T, Kanzaki R, Minami M, et al. Chest tube management in patients undergoing lobectomy. J Thorac Dis. 2018;10(12):6432–5. doi: 10.21037/jtd.2018.11.47 30746183 PMC6344739

[8] Bassi M, Mottola E, Mantovani S, Amore D, Pagini A, Diso D, et al. Coaxial drainage versus standard chest tube after pulmonary lobectomy: a randomized controlled study. Curr Oncol. 2022;29(7):4455–63. doi: 10.3390/curroncol29070354 35877214 PMC9317584

[9] Li P, Li S, Che G. Role of chest tube drainage in physical function after thoracoscopic lung resection. J Thorac Dis. 2019; 48:1947–50. 10.21037/jtd.2019.08.26 PMC6783774 31632794

[10] You J, Zhang H, Li W, Dai N, Zheng Z. Single versus double chest drains after pulmonary lobectomy: a systematic review and meta-analysis. World J Surg Oncol. 2020;18(1):175. doi: 10.1186/s12957-020-01945-1 32690055 PMC7372892

[11] Batchelor TJP. Enhanced recovery after surgery and chest tube management. J Thorac Dis. 2023;15(2):901–8. doi: 10.21037/jtd-22-1373 36910059 PMC9992626

[12] Pawelczyk K, Marciniak M, Kacprzak G, Kolodziej J. One or two drains after lobectomy? A comparison of both methods in the immediate postoperative period. Thorac Cardiovasc Surg. 2007;55(5):313–6. doi: 10.1055/s-2007-964930 17629862

[13] Wallwork K, Belcher E. Post-lobectomy airways complications. Shanghai Chest. 2021;5:18–18. doi: 10.21037/shc.2020.03.09

[14] Alex J, Ansari J, Bahalkar P, Agarwala S, Rehman MU, Saleh A, et al. Comparison of the immediate postoperative outcome of using the conventional two drains versus a single drain after lobectomy. Ann Thorac Surg. 2003;76(4):1046–9. doi: 10.1016/s0003-4975(03)00884-1 14529982

[15] Okur E, Baysungur V, Tezel C, Sevilgen G, Ergene G, Gokce M, et al. Comparison of the single or double chest tube applications after pulmonary lobectomies. Eur J Cardiothorac Surg. 2009;35(1):32–5; discussion 35-6. doi: 10.1016/j.ejcts.2008.09.009 18929492

[16] Gómez-Caro A, Roca MJ, Torres J, Cascales P, Terol E, Castañer J, et al. Successful use of a single chest drain postlobectomy instead of two classical drains: a randomized study. Eur J Cardiothorac Surg. 2006;29(4):562–6. doi: 10.1016/j.ejcts.2006.01.019 16495069

[17] Tanaka M, Sagawa M, Usuda K, Machida Y, Ueno M, Motono N, et al. Postoperative drainage with one chest tube is appropriate for pulmonary lobectomy: a randomized trial. Tohoku J Exp Med. 2014;232(1):55–61. doi: 10.1620/tjem.232.55 24492628

